# Rare Presentation of a Gastrointestinal Stromal Tumor Encapsulating the Spleen: A Case Report

**DOI:** 10.7759/cureus.37125

**Published:** 2023-04-04

**Authors:** Ashley C Calise, Zoe Leuthner, Zachary Griffith

**Affiliations:** 1 General Surgery, Alabama College of Osteopathic Medicine, Dothan, USA; 2 General Surgery, North Alabama Medical Center, Florence, USA

**Keywords:** c-kit, splenic encapsulation, splenic metastasis, gleevec, imatinib, gastrointestinal stromal tumor (gist)

## Abstract

Gastrointestinal stromal tumors (GISTs) are rare tumors of the digestive tract, often found incidentally on imaging. Although these tumors possess malignant potential, splenic encapsulation has not yet been described in the literature. A 74-year-old male fell and suffered blunt abdominal trauma followed by a 20-pound weight loss, early satiety, and left-sided abdominal pain. Computed tomography (CT) imaging showed splenomegaly with gastric compression. At the time of surgery, it was felt that this was a neoplastic process. He underwent a subsequent splenectomy and en bloc wedge gastrectomy. Further analysis revealed a GIST, of gastric origin, encapsulating the spleen and invading the diaphragm. Specimen stained strongly positive for the cluster of differentiation (CD) 117 mutation. Following recovery from the operation, the patient was started on Imatinib (Gleevec) therapy and will continue treatment for five years. Splenic metastasis and contiguous spread are rare sequelae of GISTs. While these tumors hold the potential for metastasis, the primary locations are the liver and peritoneum. This case illustrates the importance of considering malignancy as a possible underlying etiology when presented with an apparent splenic hematoma and abdominal pain. Since this patient possessed the CD117 mutation, Imatinib is an appropriate therapeutic choice in addition to surgical resection of the neoplasm.

## Introduction

Gastrointestinal stromal tumors (GISTs) refer to a group of tumors located within the digestive tract with malignant potential [1]. Typically found in the upper gastrointestinal (GI) tract, these tumors arise from pluripotent mesenchymal stem cells that then differentiate into the interstitial cells of Cajal [2]. Common locations for GISTs include primarily the stomach (60%) and small intestine (30%) with rarer incidences in the colon, mesentery, rectum, and esophagus. Gastric GISTs are often found incidentally by endoscopy but common presenting symptoms can include early satiety, abdominal pain, or GI bleeding [3].

GISTs derive oncogenic potential from gain of function mutations involving receptor tyrosine kinases (RTK), with 80% arising from mutations of c-Kit protooncogene (cKIT) and 5-10% from platelet-derived growth factor receptor alpha (PDGFRA), both of which result in constitutively active RTKs and aberrant downstream signaling [3]. These tumors are thus characterized by a positive immunohistochemistry stain for the cluster of differentiation (CD) 117 protein, a c-Kit gene product, as well as CD34 staining which can differentiate GISTs from other GI neoplasms [4]. However, the lack of such mutation does not exclude GIST as the diagnosis. Morphologically, GISTs feature spindle cells and/or epithelioid cells [5].

The recommended treatment for GISTs is surgical resection with negative margins. Management of metastatic GISTs includes additional treatment with imatinib mesylate or Gleevec. The presence of cKIT or PDGFRA mutations is a strong predictor of tumor response to Imatinib [3]. 

Our case highlights an atypical presentation of a GIST that originated in the stomach and spread contiguously to encapsulate the spleen and invade the diaphragm. Splenic involvement in GISTs is rare and to our knowledge, no other reports of similar presentations have been described in the literature. 

## Case presentation

The patient is a 74-year-old male who presented as a referral from his primary care provider complaining of abdominal pain. He had suffered a fall seven weeks prior, where he landed on the left side of his chest and abdomen. Following the fall, he underwent rib detail radiographs and it was determined that he did not have any rib fractures. Since his primary evaluation, his pain had increased and his appetite had decreased, resulting in a 20-pound reported weight loss. The pain was predominantly located in the left upper quadrant and radiated down his left side.

Past medical history included benign prostatic hypertrophy (BPH), osteoarthritis, and gastroesophageal reflux disease (GERD). His only medication was finasteride, 5 mg daily for BPH. Surgical history included four transurethral resections of the prostate (TURP) procedures. He denied alcohol and tobacco use.

**Figure 1:**
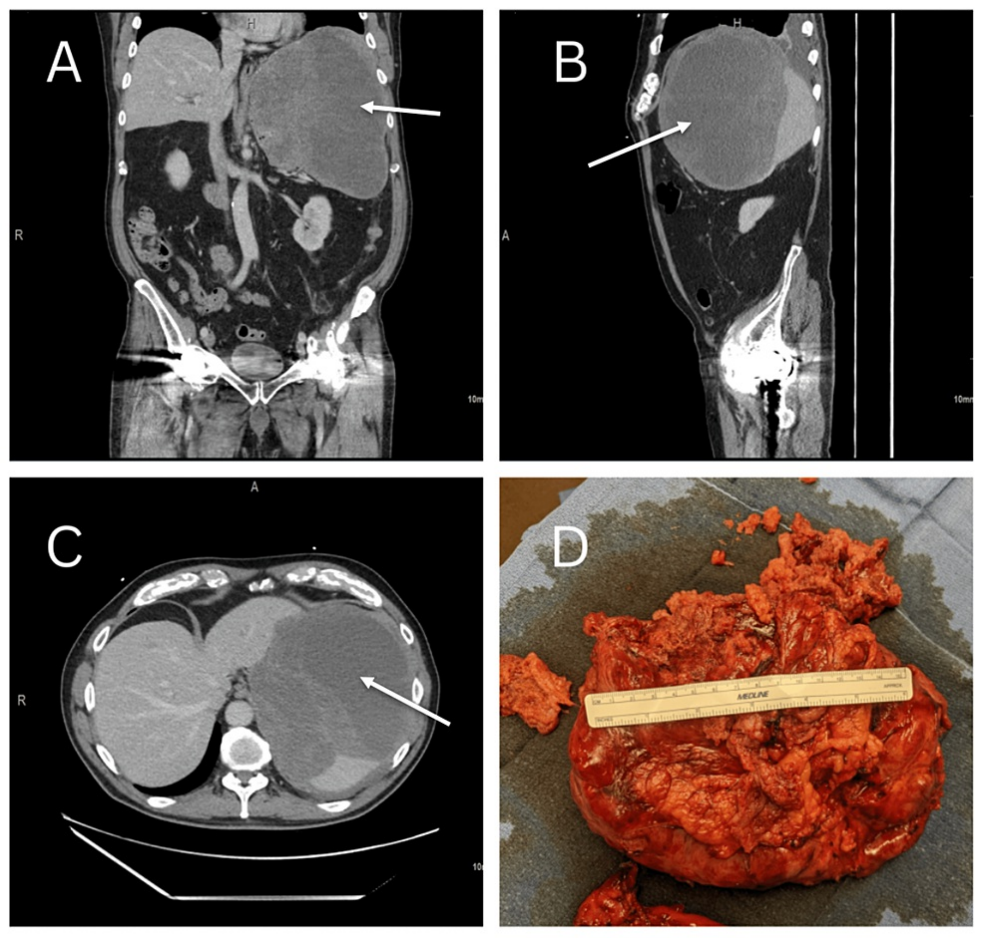
Preoperative imaging, arrows pointing to enlarged spleen. (A) Coronal view. (B) Sagittal view. (C) Axial view. (D) Gross specimen: stomach, tumor, and spleen Images published with permission.

The patient’s primary care physician obtained computed tomography (CT) of the abdomen with intravenous (IV) contrast. CT showed a large complex splenic mass favored to be a hematoma or neoplasm measuring 22 x 16 x 16 cm containing multiple internal septations and a thick rim. These images are seen in Figure 1. Additionally, compression of adjacent structures was seen, including compression of the stomach, inferior displacement of the left kidney (Figure 1C), and compression of the left hemidiaphragm (Figure 1B). Also noted was a left-sided pleural effusion. Surgical consultation was sought and advised a splenectomy; an open approach was recommended given the size, presence of symptoms, and compression of surrounding structures. After a discussion with the patient and his family, the decision to pursue surgery was reached. The patient was vaccinated, he received the haemophilus B and meningococcal conjugate vaccines, and was typed and screened in preparation. 

At surgery, the abdomen was entered via a left subcostal incision; once the abdomen was entered, the spleen was immediately visualized. There were noted to be dense adhesions of the omentum to the surface of the spleen. The dissection was carried out and it became clear that there was no discrete plane between the stomach and the spleen so en bloc wedge gastrectomy was performed. Further, the plane between the splenic capsule and the diaphragm had been completely obliterated, resulting in a portion of the central tendon and muscle being taken with the specimen. During the process of mobilization, the presumed splenic hematoma was entered. The CellSaver device (Haemonetics Corporation, Boston, Massachusetts, United States) was employed during the surgery. The spleen displayed characteristics consistent with malignancy with several areas of necrosis. All residual areas of splenic tissue were taken individually with the LigaSure (Medtronic plc, Dublin, Ireland). The gastrostomy was closed with a GI anastomosis (GIA) stapler. The diaphragmatic lacerations were also repaired with 0-Ethibond sutures (Ethicon, Inc., Raritan, New Jersey, United States). A #10 Jackson-Pratt (JP) drain was placed in the splenophrenic fossa. The estimated blood loss was 3.5 liters. The patient received seven units of packed red cells, two units of fresh frozen plasma, and one unit of platelets. Following the operation, the patient was transferred to the intensive care unit (ICU) in stable condition. 

**Figure 2:**
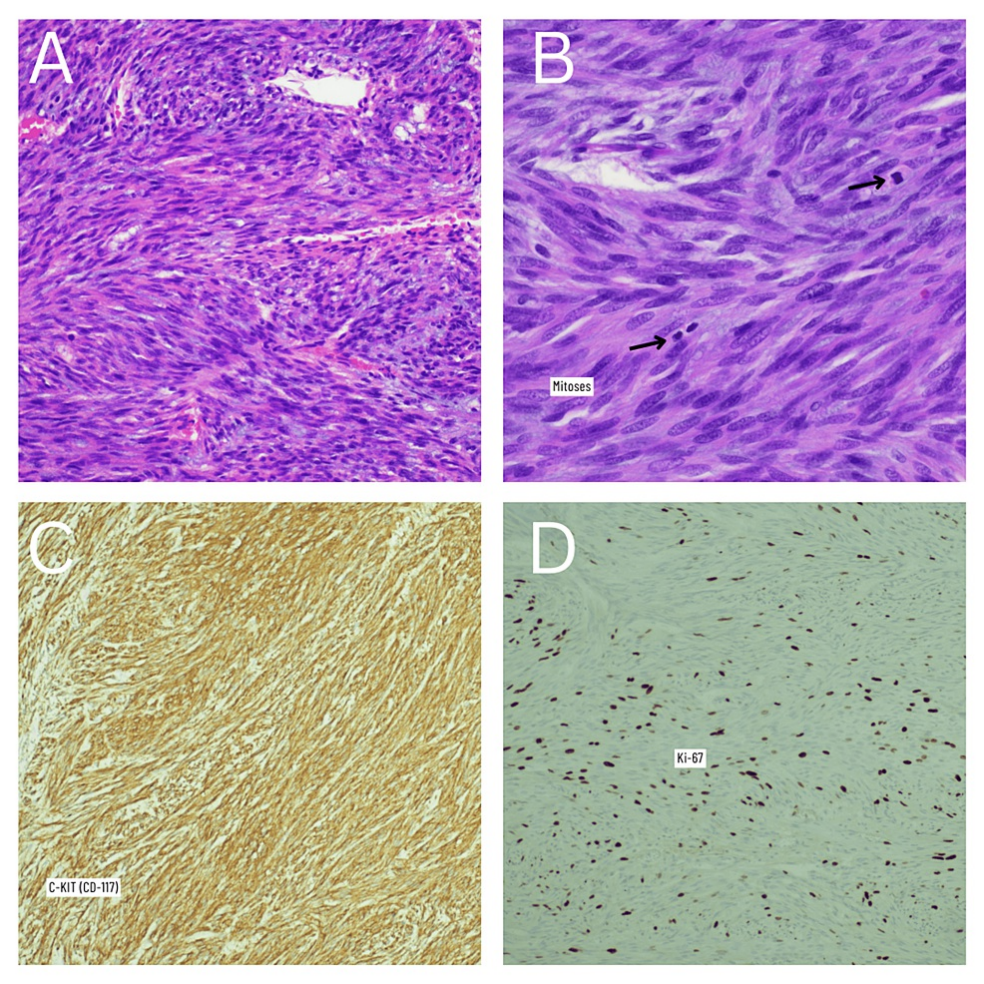
Histopathology images and immunohistochemistry staining. (A) Hematoxylin & Eosin staining, gastrointestinal stromal tumor; spindle cells. (B) Hematoxylin & Eosin staining showing mitoses. (C) Positive C-KIT (CD-117) immunohistochemistry stain. (D) Ki-67 immunohistochemistry staining showing proliferation.

The pathology report indicated that the specimen removed was a nodular specimen weighing 1,524 grams and measuring 24.0 x 20.0 x 7.5 cm (Figure 1D). Upon further gross examination, the splenic capsule appeared to be partially torn. Gross sectioning of the spleen concluded that the spleen itself was 15.5 x 8.0 x 2.6 cm and weighed 154 grams. Additionally, the splenic parenchyma was largely intact with no suspicious lesions or nodules identified. It was concluded that the mass was perisplenic with areas of peripheral and central necrosis. The gastric wedge was appreciated and measured 11.0 x 3.0 x 1.5 cm; further evaluation revealed the gastric tissue contained a GIST, spindle cell type, located in the muscular wall with negative margins (Figure 2A). The microscopic evaluation concluded the tumor originated in the stomach and spread contiguously to encapsulate the spleen with additional diaphragmatic attachments. The size, mitotic count, and KI-67 proliferation index (24%) conveyed that the GIST was in the “High rate of progression” group (Figure 2). 

The patient was in the ICU for five days following surgery. As expected, the patient exhibited thrombocytosis following surgery, reaching one million before discharge to rehab. On postoperative day seven, the patient received a Hematology/Oncology consult. Following discharge and recovery, the patient was started on Imatinib (Gleevec) therapy after outpatient CT imaging. He will continue treatment with Imatinib for five years. On surgical follow-up, the patient reported he had returned to his normal activities and is doing well. He also reported a resolution of early satiety and improvement in his dyspeptic symptoms. 

## Discussion

GISTs are neoplasms found in the digestive tract that possess the potential for malignant behavior [1]. The most frequent site of metastasis is the liver, but another common site is the peritoneum [4].

One of the major diagnostic criteria of GISTs is the expression of the cKit (CD117) antigen. Additionally, a GIST may also stain CD34 positively. Around 60-70% of GISTs contain c-kit mutations [6]. Identification of such mutations has become more important when evaluating a GIST due to the response to a Kit-selective tyrosine kinase inhibitor, Imatinib (Gleevec) [6]. When predicting malignancy, tumors that are larger than 5 cm and contain more than five mitotically active cells per 50 high-powered fields (HPF) are suspicious for malignancy. The tumor from our patient stained strongly positive for CD117 (Figure 2C) as well as CD34. There were 12 mitotic figures per 50 HPF. Initial tumor staging was pT4/NX.

Splenic involvement in GISTs is exceedingly rare, with few reports describing metastasis or contiguous spread to the spleen. One case report described metastasis to the spleen as well as the liver and greater omentum in a patient with a GIST recurrence after previous resection; however, the lesion was found to invade splenic parenchyma. [7]. Regarding the involvement of the pancreas and spleen, one retrospective review stated that of 37 patients who underwent resection of GISTs larger than 10 cm, 15 (41%) underwent distal pancreatectomy and splenectomy, although, only approximately one quarter demonstrated invasion of resected organs [8]. Interestingly, none of the cases found during the literature review reported complete GIST encapsulation of the spleen, without underlying invasion, as in our case.

## Conclusions

We presented a case of a GIST of the stomach with contiguous growth that encapsulated the spleen. Surgeons should be aware of atypical presentations of GISTs; these tumors can spread contiguously to surrounding structures, including the spleen, and present similarly to splenic hematomas with nonspecific CT findings. Early recognition and appropriate intervention are imperative to the evaluation and management of such cases.
